# Spatio-temporal numerical modeling of stochastic predator-prey model

**DOI:** 10.1038/s41598-023-28324-6

**Published:** 2023-02-03

**Authors:** Muhammad W. Yasin, Nauman Ahmed, Muhammad S. Iqbal, Ali Raza, Muhammad Rafiq, Elsayed Mohamed Tag eldin, Ilyas Khan

**Affiliations:** 1 Department of Mathematics and Statistics, The University of Lahore, Lahore, Pakistan; 2 Department of Mathematics, University of Narowal, Narowal, Pakistan; 3 Department of Humanities and Basic Science, MCS, National University of Science and Technology, Islamabad, Pakistan; 4 Department of Mathematics, Govt. Maulana Zafar Ali Khan Graduate College Wazirabad, Punjab Higher Education Department (PHED), Lahore, 54000 Pakistan; 5 Department of Mathematics, Faculty of Science and Technology, University of the Central Punjab, Lahore, Pakistan; 6 Department of Mathematics, Near East University, Mathematics Research Center, Near East Boulevard, PC: 99138 Nicosia/Mersin 10, Turkey; 7 Faculty of Engineering and Technology, Future University in Egypt, 11835 New Cairo, Egypt; 8 Department of Mathematics, College of Science Al-Zulfi, Majmaah University, 11952 Al-Majmaah, Saudi Arabia; 9 Department of Computer Science and Mathematics, Lebanese American University, Beirut, Lebanon

**Keywords:** Computational biology and bioinformatics, Diseases

## Abstract

In this article, the ratio-dependent prey-predator system perturbed with time noise is numerically investigated. It relates to the population densities of the prey and predator in an ecological system. The initial prey-predator models only depend on the time and a couple of the differential equations. We are considering a model where the prey-predator interaction is influenced by both space and time and the need for a coupled nonlinear partial differential equation with the effect of the random behavior of the environment. The existence of the solutions is guaranteed by using Schauder’s fixed point theorem. The computation of the underlying model is carried out by two schemes. The proposed stochastic forward Euler scheme is conditionally stable and consistent with the system of the equations. The proposed stochastic non-standard finite difference scheme is unconditionally stable and consistent with the system of the equations. The graphical behavior of a test problem for different values of the parameters is shown which depicts the efficacy of the schemes. Our numerical results will help the researchers to consider the effect of the noise on the prey-predator model.

## Introduction

The physical systems are part of nature. The scientists tried different techniques to understand these phenomena and developed various physical models by using a set of equations. They used numerical and analytical methods for the solutions of such models. So, that they can understand the phenomena and decide for the betterment of human life. The life of man has links with living organisms and nonliving things and ecosystems are made of such organisms and substances. There is constant interaction between and among ecosystem components and it is an interesting phenomenon how ecosystem population dynamics evolve. When population dynamics are simply observed over time, they are referred to as temporal dynamics and when they are observed over both time and space, they are referred to as spatiotemporal dynamics. The population dynamics produce a lot of tough issues for the researchers^1–3^. The prey-predator interaction has gained the most attention among the ecosystem interactions and predator has a severe effect on the prey-predator population. Predation can alter the population of the prey to extinction and then the extinction of predators.

The first prey-predator interaction was derived independently by Lotka^4^ and Volterra^5^ and it is known as the Lotka-Volterra prey-predator model. Various researchers worked on the different aspects of the prey-predator model and added some terms in the models that explain the versatility of the models as compared with the Lotka–Volterra prey-predator model. There are two types of prey-predator models, one is prey-dependent, and the second is a ratio-dependent model. In terms of geometry, the prey-dependent model has a vertical predator isocline, while the ratio-dependent model has a tilted one^6^. There are two main predictions made by the ratio-dependent prey-predator models:(i) along a gradient of enrichment, equilibrium abundances are positively linked^7^ (ii)the “paradox of enrichment”^8^ either entirely extinct or enrichment is connected to stability. In^9^, Maiti et al. considered the following ratio-dependent model

1 $$\begin{aligned} \frac{dx}{dt}=x(a-bx)-\frac{cxy}{my+x}, \qquad \frac{dy}{dt}=-dy + \frac{fxy}{my+x}, \end{aligned}$$

where *y*(*t*) and *x*(*t*) represent the population density of the predator and prey at any time t. They discuss the various aspects of the model such as uniform boundedness, stability, Hopf bifurcation, etc. In this model population densities only depends on time and a couple of the differential equations. In a natural system, either the predator or the prey may move from one area to another for a variety of reasons. In this scenario, the prey-predator interaction is influenced by both space and time and the need for a coupled nonlinear partial differential equation. If such models are under investigation and also considering the effect of the fluctuation of the environment then stochastic models are preferable.

The stochastic version of the ratio-dependent prey-predator model is taken as

2 $$\begin{aligned} \frac{\partial P}{\partial t}= & {} \sigma _1\frac{\partial ^2 P}{\partial x^2}+ P(a-bP)-\frac{cPQ}{mQ+P}+\eta _1 P \dot{B}_1(t), \end{aligned}$$

3 $$\begin{aligned} \frac{\partial Q}{\partial t}= & {} \sigma _2 \frac{\partial ^2 Q}{\partial x^2}-dQ +\frac{fPQ}{mQ+P}+\eta _2 Q \dot{B}_2(t), \end{aligned}$$

having initial

4 $$\begin{aligned} P(x,0)= & {} \alpha (x),\nonumber \\ Q(x,0)= & {} \beta (x), \end{aligned}$$

and homogeneous Neumann boundary conditions. Where *P*(*x*, *t*) and *Q*(*x*, *t*) represent the population densities of the prey and predator at any point (*x*, *t*) respectively. Here $d>0$ is the death rate of the predator, $\frac{a}{b}>0$ is the carrying capacity of the prey, and m, f, c, a are positive constants that denote half capturing saturation constant, conversion rate, capturing rate, and prey intrinsic growth rate respectively. The diffusion coefficients are $\sigma _1>0$ and $\sigma _2>0$. The $B_1(t)$ and $B_2(t)$ are the one-dimensional standard Wiener processes such that the $\dot{B}_1(t)$ and $\dot{B}_2(t)$ are the Gaussian distribution with zero mean^10^,$\eta _1$ and $\eta _2$ are the noisy strengths which are the Borel functions.

Dealing with stochastic partial differential equations numerically is not a simple task and it becomes more difficult when it has nonlinear terms. Various researcher is working on the numerical solution of the SPDEs. Iqbal et al. considered the stochastic Newell-Whitehead-Segel equation. They discussed the existence results, derived the numerical approximation by two finite difference schemes, and proved the consistency and stability of the schemes in man square sense^11^. The authors used the multiple scale method for the numerical solutions of the SPDEs having quadratic nonlinearities^12^. Allen et al. worked numerically on the linear elliptic and parabolic SPDEs under the influence of white noise by finite difference and element methods. They showed that both methods have the same order of accuracy but the different method is not as computationally efficient as the finite element method^13^. The authors obtained the approximation of the linear SPDEs with special additive noise. The error analysis and convergence analysis of the standard finite difference and element methods are discussed. The impacts of noise on approximation accuracy are explained^14^.

Some researchers worked on the consistency and stability of the schemes as well. Namjoo et al worked on the numerical approximation of the linear SPDEs of the It$\hat{o}$ type. They showed the consistency, stability, and convergence of the finite difference scheme^15^. In^16^, the authors found the numerical computing of hyperbolic SPDEs with finite difference methods and discussed the stability, consistency, and convergence of the scheme. Kruse worked on the computational approximation of semi-linear SPDEs by using the Milstein–Galerkin finite element scheme and discussed the error analysis of the scheme^17^. Sohalay worked on the numerical solutions of parabolic SPDEs with finite difference methods and derived the condition of convergence in mean square sense^18^.

Belabbas et al. worked on the stochastic prey-predator model under the influence of multiplicative noise with a protection zone for the prey. They discuss the different aspects of the models such as the existence, uniqueness of the global positive solutions, and boundedness. The conditions for the extinction and persistence of two species are derived^19^. The authors worked on the existence of the solutions for the class of the stochastic differential equation^20^. Souna et al. considered the prey-predator model and applied the linear stability analysis to gain the conditions for the Turing-driven instability and Hopf bifurcation^21^. More work on prey-predator, one may see^22–24^.

The prey-predator models are population dynamical models and necessarily the solutions must be positive. We have applied two techniques for the numerical solutions of the prey-predator model. One technique fails to preserve the convergent and positive behavior while the other preserves the positivity and convergent toward the steady states. One of the strongest motivations for considering this model is to ensure the application and construction of the numerical scheme which provides positive solutions as per the requirement of the underlying model because the under consideration model is a population dynamical model and the population may attain minimum value zero and can never be negative. So many solutions are preferred which preserve the positivity and bounded behavior for the whole domain. The results of the stochastic non-standard finite difference scheme are aligned with the actual steady states which are the positive steady states. The diffusion process is hardly considered for the prey-predator models and diffusion is a basic phenomenon in the population dynamical models because they interact with each other and diffuse with a certain diffusion rate. If we are considering the very smallest organism population models, they are not continuous in the usual sense. The microorganisms can mix and produce random behavior. So, it is quite better to consider the continuous model with a random effect. Such random behavior is observed in every physical phenomenon at a certain level. So we incorporate diffusion as well as random behavior in the prey-predator model.

## Existence and regularity analysis

The coupled system (2) and (3) can be inverted in the form of the following Volterra type integral equations;

5 $$\begin{aligned} {\textbf {P}}(x,t)= & {} \alpha (x) + \int _0^t\bigg ( \sigma _1\frac{\partial ^2 P}{\partial x^2}+ P(a-bP)-\frac{cPQ}{mQ+P}+\eta _1 P \dot{B}_1(t) \bigg )ds, \end{aligned}$$

6 $$\begin{aligned} {\textbf {Q}}(x,t)= & {} \beta (x) + \int _0^t\bigg ( \sigma _2 \frac{\partial ^2 Q}{\partial x^2}-dQ +\frac{fPQ}{mQ+P}+\eta _2 Q \dot{B}_2(t)\bigg )ds, \end{aligned}$$

where *P* and *Q* are positive, being the population densities and they are the function of space and time i.e., *P*(*x*, *t*) and *Q*(*x*, *t*).

The goal of the current section is to guarantee the existence of the vector $(P^*,Q^*)$ being at least one solution of the system (2) and (3). The Eqs. (5) and (6) are assumed as the fixed point operator equations. For existence, we choose the space of continuous functions *C* equipped with supremum norm. Subsequently, we have to see the existence of the solution in a closed, convex, and bounded subset of Banach space defined as

7 $$\begin{aligned} B_r(\Theta )=[P,Q; P,Q \in C[0,\rho ],||P||\le r, ||Q||\le r ], \end{aligned}$$

where $\Theta$ is the zero elements of the function space *C*. The Schauder fixed point theorem will be used leading to the following two conditions to be verified. (i) ${\textbf {P,Q}}:B_r(\Theta ) \rightarrow B_r(\Theta )$, (ii) ${\textbf {P}}(B_r(\Theta ))$ and ${\textbf {Q}} (B_r(\Theta ))$ relatively compact. To ensure (i), we take the norm of an Eqs. (5), and (6)

$$\begin{aligned} ||{\textbf {P}}||= & {} ||\alpha (x)|| + \int _0^t\bigg ( \sigma _1||P_{xx}||+ a||P||+b||P||^2+kc||P||||Q||+\eta _1 ||P|| ||\dot{B}_1(t)|| \bigg )ds,\\ ||{\textbf {Q)}}||= & {} ||\beta (x)|| + \int _0^t\bigg ( \sigma _2 ||Q_{xx}||+d||Q|| +kf||P||||Q||+\eta _2 ||Q|| ||\dot{B}_2(t)||\bigg )ds, \end{aligned}$$

where $||\frac{1}{mQ+P}||\le k$, is true when P and Q are positive functions. Suppose $||\dot{B}_1(t)||=||\dot{B}_2(t)||=H$, are the bounded noise, also $||P_{xx}||\le k_1$, $||Q_{xx}||\le k_2$,

8 $$\begin{aligned}{} & {} D_1+\rho (\sigma _1 k_1+ a r+ b r^2+kfr^2+ \eta _1M r) \le r, \end{aligned}$$

9 $$\begin{aligned}{} & {} D_2+\rho (\sigma _2 k_2+ d r+ kfr^2+ \eta _2M r )\le r, \end{aligned}$$

10 $$\begin{aligned}{} & {} \rho ^*\le \min \bigg (\frac{r-D_1}{\sigma _1 k_1+ a r+ b r^2+kfr^2+ \eta _1M r}, \frac{r-D_2}{\sigma _2 k_2+ d r+ kfr^2+ \eta _2M r }\bigg ), \end{aligned}$$

condition (10) is important as it serves for the length of the interval $[0, \rho ]$.

Now, it is remaining to show that $({\textbf {P, Q}}): B_r(\Theta )$ is relatively compact. For that we have two families ${\textbf {P}}_i, {\textbf {Q}}_i$ as images for pre-images $P_i,Q_i$ and we see that the difference $||{\textbf {P}}_i(t)-{\textbf {P}}_i(t^*)||$ and $||{\textbf {Q}}_i(t)-{\textbf {Q}}_i(t^*)||$ approaches to zero as $t\rightarrow t^*$ i.e., easy calculation will show that ${\textbf {P}}_i$, and ${\textbf {Q}}_i$ are equi-continuous family of operators by the well known Arzela-Ascoli theorem there exist two uniformly convergent subfamilies $P_i$, and $Q_i$. So, ${\textbf {P}}(B_r(\Theta ))$ and ${\textbf {Q}}(B_r(\Theta ))$ are relatively compact. Thus there exist at least one fixed point vector $(P^*,Q^*)$ which is the solution of the system (2) and (3). We have the following result,

### Theorem 1

If P(x,t) and Q(x,t) are twice continuously differentiable function and $\alpha (x),\beta (x), \sigma _1,\sigma _2,a,b,cd,f,\eta _1,\eta _2$ are bounded function then the coupled system (2) and (3) has solutions by Schauder fixed point theorem in (7) and the obtained solutionis continuous in $[0, \rho ^*]$, where $\rho ^*$ is defined in (10).

## Numerical schemes

The proposed stochastic forward Euler scheme (proposed scheme-I) for Eqs. (2) and (3) is given below

11 $$\begin{aligned} P_m^{k+1}= & {} D_1 (P_{m+1}^k+P_{m-1}^k)+(1-2D_1)P_m^k+\Delta \tau P_m^k(a-b P_m^k) -c \Delta \tau \frac{P_m^kQ_m^k}{m Q_m^k+P_m^k}+ \eta _1 P_m^k \left( B_1^{(k+1)\Delta \tau }-B_1^{k\Delta \tau }\right) , \end{aligned}$$

12 $$\begin{aligned} Q_m^{k+1}= & {} D_2 (Q_{m+1}^k+Q_{m-1}^k)+(1-2D_2- d \Delta \tau )Q_m^k +f \Delta \tau \frac{P_m^kQ_m^k}{m Q_m^k+P_m^k}+ \eta _2 Q_m^k \left( B_2^{(k+1)\Delta \tau }-B_2^{k\Delta \tau }\right) , \end{aligned}$$

where $\Delta \tau$ and *h* are time and space stepsizes and $D_1=\frac{\Delta \tau \sigma _1}{h^2}$ and $D_2=\frac{\Delta \tau \sigma _2}{h^2}$.

Mickens proposed a finite difference scheme that preserves the positivity of the solution. The proposed stochastic non-standard finite difference scheme (proposed scheme-II) for Eqs. (2) and (3) is given below

13 $$\begin{aligned} P_m^{k+1}= & {} \frac{D_1 (P_{m+1}^k+P_{m-1}^k)+(1+a \Delta \tau )P_m^k+ \eta _1 P_m^k(B_1^{(k+1)\Delta \tau }-B_1^{k\Delta \tau })}{1+2D_1+b \Delta \tau P_m^k +c\Delta \tau \frac{Q_m^k}{m Q_m^k+P_m^k}}, \end{aligned}$$

14 $$\begin{aligned} Q_m^{k+1}= & {} \frac{D_2 (Q_{m+1}^k+Q_{m-1}^k)+f \Delta \tau \frac{P_m^kQ_m^k}{m Q_m^k+P_m^k}+ \eta _2 Q_m^k \left( B_2^{(k+1)\Delta \Delta \tau }-B_2^{k\Delta \tau }\right) }{1+2D_2+ d \Delta \tau }. \end{aligned}$$

### Consistency of a scheme

#### Definition 1

^25–27^. A stochastic finite difference (SFD) scheme $L|_{r,s}U|_{r,s}=G|_{r,s}$ is consistent with stochastic partial differential equation $LU=G$ at a point (*x*, *t*), if there is any continuously differentiable function $\Psi =\Psi (x,t)$ then

15 $$\begin{aligned} E|| (L\Psi -G)|_{r,s}-[L|_{r,s}\Psi |_{(r \Delta x,s\Delta t)}-G|_{r,s}]||^{2} \rightarrow 0\quad , \end{aligned}$$

as $$ \Delta x \rightarrow 0, \Delta t \rightarrow 0$$ and $$(r \Delta x,(s+1) \Delta t)\rightarrow (x, t)$$.

### Von-Neumann analysis

In this technique $P_{m}^{k}$ is taken as follow

16 $$\begin{aligned} P_{m}^{k}=\frac{1}{\sqrt{2\pi }}\int _{\frac{-\pi }{\Delta x}}^{\frac{\pi }{\Delta x}} e^{\iota m\Delta x \eta } \hat{P}^{k}_{m}(\eta )d(\eta ), \end{aligned}$$

and $\hat{P}^k_m$ is defined as

$$\begin{aligned} \hat{P}^{k}_{m}=\frac{1}{\sqrt{2\pi }}\sum _{-\infty }^{\infty }e^{-\iota m\Delta x \eta }{P}_{m}^{k}\Delta x. \end{aligned}$$

Here, $\eta$ is a variable, and by putting values in the given PDE

17 $$\begin{aligned} \hat{P}^{k+1}_{m}(\eta )=\hat{P}^{k}_{m}(\eta )g(\eta \Delta x, \Delta x, \Delta t). \end{aligned}$$

The following is the necessary and sufficient condition for this method^28^.

18 $$\begin{aligned} E\left| g(\eta \Delta x, \Delta x, \Delta t)\right| ^2\le 1+\chi \Delta t, \end{aligned}$$

where $\chi$ is a constant.

### Consistency of proposed scheme-I

The consistent results of the schemes are established in the mean square sense.

#### Theorem 2

The proposed SFE scheme for *P*, *Q* in (11,12) is consistent with (2,3), in mean square sense.

#### Proof

Suppose that *P*(*x*, *t*) and *Q*(*x*, *t*) are smooth functions and by using the $L(f) = \int ^{(n+1)\Delta \tau }_{n\Delta \tau } {f} ds$ on Eq. (2). We get

19 $$\begin{aligned} L(P)|_{m,k}&= P(m h, (k+1)\Delta \tau )-P(mh, k\Delta \tau )- \sigma _1 \int ^{(k+1)\Delta \tau }_{k\Delta \tau }P_{xx}(m h,u) du- \int ^{(k+1)\Delta \tau }_{k\Delta \tau }P(m h,u)(a-bP (mh,u)) du \nonumber \\{} &\quad {} +c \int ^{(k+1)\Delta \tau }_{k\Delta \tau }\frac{Q(m h,u)P(m h,u)}{P(m h,u)+mQ(m h,u)}du- \eta _1 \int ^{(k+1)\Delta \tau }_{k\Delta \tau }P(m h,u) d B_1(u). \end{aligned}$$

By using the proposed SFE scheme on Eq. (2)

20 $$\begin{aligned} L|_{m,k}(P)&= P(m h, (k+1)\Delta \tau )-P(mh, k\Delta \tau )-\sigma _1 \Delta \tau \frac{P((m+1)h,k\Delta \tau )-2P(m h,k\Delta \tau )+P((m-1)h,k\Delta \tau )}{h^2} \nonumber \\{} &\quad {} - \Delta \tau P(m h,k\Delta \tau )(a-b P(m h,k\Delta \tau ))+ c\Delta \tau \frac{Q(m h, k \Delta \tau )P(m h,k \Delta \tau )}{P(m h,k\Delta \tau )+m Q(m h,k\Delta \tau )}\nonumber \\{} &\quad {} - \eta _1 P(m h,k\Delta \tau ) (B_1^{(k+1)\Delta \tau }-B_1^{k\Delta \tau }). \end{aligned}$$

Equations (19) and (20) takes the form

$$\begin{aligned}{} & {} E|L(P)|_{m,k}-L|_{m,k} P|^2 \le 4\sigma _1^2 E \Bigg|\int ^{(k+1)\Delta \tau }_{k\Delta \tau }(-P_{xx}(m h,u)\\{} & {} \quad +\frac{P((m+1) h,k\Delta \tau )-2P(m h,k\Delta \tau )+P((m-1)h,k\Delta \tau )}{h^2})ds\Bigg|^2\\{} & {} \quad +4 E \Bigg|\int ^{(k+1)\Delta \tau }_{k\Delta \tau }\left( -P(m h,u)(a-bP(m h,u))+P(m h,k\Delta \tau )(a -bP(m h,k\Delta \tau ))\right) du\Bigg|^2\\{} & {} \quad + 4c^2E\Bigg|\int ^{(k+1)\Delta \tau }_{k\Delta \tau }\left( \frac{Q(m h,u)P(m h,u)}{P(m h,u)+mQ(m h,u)}- \frac{Q(m h, k \Delta \tau )P(m h,k \Delta \tau )}{P(m h,k\Delta \tau )+m Q(m h,k\Delta \tau )}\right) du\Bigg|^2 \\{} & {} \quad +4 \eta _1^2E\Bigg|\int ^{(k+1)\Delta \tau }_{k\Delta \tau }\left( -P(m h,u)+P(m h,k\Delta \tau ) \right) d B_1(u)\Bigg|^2. \end{aligned}$$

By using the symmetry property of the Itô’s integral

$$\begin{aligned}{} & {} E|L(P)|_{m,k}-L|_{m,k} P|^2 \le 4\sigma _1^2 E \Bigg|\int ^{(k+1)\Delta \tau }_{k\Delta \tau }(-P_{xx}(m h,u)\\{} & {} \quad +\frac{P((m+1) h,k\Delta \tau )-2P(m h,k\Delta \tau )+P((m-1)h,k\Delta \tau )}{h^2})ds\Bigg|^2\\{} & {} \quad +4 E \Bigg|\int ^{(k+1)\Delta \tau }_{k\Delta \tau }\left( -P(m h,u)(a-bP(m h,u))+P(m h,k\Delta \tau )(a -bP(m h,k\Delta \tau ))\right) du\Bigg|^2\\{} & {} \quad + 4c^2E\Bigg|\int ^{(k+1)\Delta \tau }_{k\Delta \tau }\left( \frac{Q(m h,u)P(m h,u)}{P(m h,u)+mQ(m h,u)}- \frac{Q(m h, k \Delta \tau )P(m h,k \Delta \tau )}{P(m h,k\Delta \tau )+m Q(m h,k\Delta \tau )}\right) du\Bigg|^2 \\{} & {} \quad +4\eta _1^2 \int ^{(k+1)\Delta \tau }_{k\Delta \tau }E\Bigg|(-P(m h,u)+P(m h,k\Delta \tau ))\Bigg|^2 du. \end{aligned}$$

$E|L(P)|_{m,k}-L|_{m,k}(P)|^2\rightarrow 0$ as $m\rightarrow \infty , q \rightarrow \infty ,$ so the proposed scheme for *P* is consistent with stochastic PDE (2).

Now, to check the consistency of (12) with SPDE (3).

21 $$\begin{aligned} L(Q)|_{m,k}= & {} Q(m h,(k+1)\Delta \tau )-Q(m h,k\Delta \tau )- \sigma _2 \int ^{(k+1)\Delta \tau }_{k\Delta \tau }Q_{xx}(m h,u) du \nonumber \\{} & {} + \int ^{(k+1)\Delta \tau }_{k\Delta \tau }d Q(m h,u)du- f \int ^{(k+1)\Delta \tau }_{k\Delta \tau }\frac{Q(m h,u)P(m h,u)}{P(m h,u)+mQ(m h,u)}du \nonumber \\{} & {} - \eta _2 \int ^{(k+1)\Delta \tau }_{k\Delta \tau }Q(m h,u) d B_2(u). \end{aligned}$$

By using the proposed SFE scheme on Eq. (3)

22 $$\begin{aligned} L|_{m,k}(Q)= & {} Q(m h,(k+1)\Delta \tau )-Q(m h,k\Delta \tau )\nonumber \\{} & {} -\sigma _2 \Delta \tau \frac{Q((m+1)h,k\Delta \tau )-2Q(m h,k\Delta \tau )+Q((m-1)h,k\Delta \tau )}{h^2} \nonumber \\{} & {} +d \Delta \tau Q(m h,k\Delta \tau )- f\Delta \tau \frac{Q(m h, k \Delta \tau )P(m h,k \Delta \tau )}{P(m h,k\Delta \tau )+m Q(m h,k\Delta \tau )}\nonumber \\{} & {} - \eta _2 Q(m h,k\Delta \tau ) (B_2^{(k+1)\Delta \tau }-B_2^{k\Delta \tau }). \end{aligned}$$

Equations (21) and (22) takes the form

$$\begin{aligned}{} & {} E|L(Q)|_{m,k}-L|_{m,k} Q|^2 \le 4\sigma _2^2 E \Bigg|\int ^{(k+1)\Delta \tau }_{k\Delta \tau }(-Q_{xx}(m h,u)\\{} & {} \quad +\frac{Q((m+1) h,k\Delta \tau )-2Q(m h,k \Delta \tau )+Q((m-1)h,k\Delta \tau )}{h^2})du\Bigg|^2\\{} & {} \quad +4 d^2 E \Bigg|\int ^{(k+1)\Delta \tau }_{k\Delta \tau }\left( Q(m h,u)-Q(m h,k\Delta \tau )\right) du\Bigg|^2\\{} & {} \quad + 4E\Bigg|\int ^{(k+1)\Delta \tau }_{k\Delta \tau }\left( \frac{-f Q(m h,u)P(m h,u)}{P(m h,u)+mQ(m h,u)}+\frac{f Q(m h, k \Delta \tau )P(m h,k \Delta \tau )}{P(m h,k\Delta \tau )+m Q(m h,k\Delta \tau )}\right) du\Bigg|^2 \\{} & {} \quad +4 \eta _2^2E\Bigg|\int ^{(k+1)\Delta \tau }_{k\Delta \tau }\left( -Q(m h,u)+Q(m h,k\Delta \tau ) \right) d B_2(u)\Bigg|^2. \end{aligned}$$

By using the symmetry property of the Itô’s integral

$$\begin{aligned}{} & {} E|L(Q)|_{m,k}-L|_{m,k} Q|^2 \le 4\sigma _2^2 E \Bigg|\int ^{(k+1)\Delta \tau }_{k\Delta \tau }(-Q{xx}(m h,u)\\{} & {} \quad +\frac{Q((m+1) h,k\Delta \tau )-2Q(m h,k \Delta \tau )+Q((m-1)h,k\Delta \tau )}{h^2})du\Bigg|^2\\{} & {} \quad +4 d^2 E \Bigg|\int ^{(k+1)\Delta \tau }_{k\Delta \tau }\left( Q(m h,u)-Q(m h,k\Delta \tau )\right) du\Bigg|^2\\{} & {} \quad + 4E\Bigg|\int ^{(k+1)\Delta \tau }_{k\Delta \tau }\left( \frac{-f Q(m h,u)P(m h,u)}{P(m h,u)+mQ(m h,u)}+\frac{f Q(m h, k \Delta \tau )P(m h,k \Delta \tau )}{P(m h,k\Delta \tau )+m Q(m h,k\Delta \tau )}\right) du\Bigg|^2 \\{} & {} \quad +4\eta _2^2 \int ^{(k+1)\Delta \tau }_{k\Delta \tau }E\Bigg|(-Q(m h,u)+Q(m h,k\Delta \tau ))\Bigg|^2 du. \end{aligned}$$

$E|L(Q)|_{m,k}-L|_{m,k} Q|^2\rightarrow 0$ as $m\rightarrow \infty , k \rightarrow \infty ,$ so the proposed scheme for *Q* is consistent with stochastic PDE (3). $\square$

### Consistency of proposed scheme-II

The consistent results of the schemes are established in the mean square sense.

#### Theorem 3

The proposed stochastic NSFD scheme for *P*, *Q* in (13,14) is consistent with (2,3), in mean square sense.

#### Proof

Suppose that *P*(*x*, *t*) and *Q*(*x*, *t*) are smooth functions and by using the $L(f) = \int ^{(k+1)\Delta \tau }_{k\Delta \tau } {f} ds$ on Eq. (2). We get

23 $$\begin{aligned} L(P)|_{m,k}= & {} P(m h, (k+1)\Delta \tau )-P(mh, k\Delta \tau )- \sigma _1 \int ^{(k+1)\Delta \tau }_{k\Delta \tau }P_{xx}(m h,u) du- \int ^{(k+1)\Delta \tau }_{k\Delta \tau }P(m h,u)(a-bP (m h,u)) du\nonumber \\{} & {} +c \int ^{(k+1)\Delta \tau }_{k\Delta \tau }\frac{Q(m h,u)P(m h,u)}{P(m h,u)+mQ(m h,u)}du- \eta _1 \int ^{(k+1)\Delta \tau }_{k\Delta \tau }P(m h,u) d B_1(u). \end{aligned}$$

By using the proposed stochastic NSFD scheme on Eq. (2)

24 $$\begin{aligned} L|_{m,k}(P)= & {} P(m h, (k+1)\Delta \tau )-P(mh, k\Delta \tau )-\sigma _1 \Delta \tau \frac{P((m+1)h,k\Delta \tau )-2P(m h,(k+1)\Delta \tau )+P((m-1)h,k\Delta \tau )}{h^2} \nonumber \\{} & {} - \Delta \tau P(m h,k\Delta \tau )(a-b P(m h,(k+1)\Delta \tau ))+ c\Delta \tau \frac{Q(m h, \Delta \tau )P(m h,(k+1) \Delta \tau )}{P(m h,k\Delta \tau )+m Q(m h,k\Delta \tau )} \nonumber \\{} & {} -\eta _1 P(m h,k\Delta \tau ) (B_1^{(k+1)\Delta \tau }-B_1^{k\Delta \tau }). \end{aligned}$$

Equations (23) and (24) takes the form

$$\begin{aligned}{} & {} E|L(P)|_{m,k}-L|_{m,k} P|^2 \le 4\sigma _1^2 E \Bigg|\int ^{(k+1)\Delta \tau }_{k\Delta \tau }(-P_{xx}(m h,u)\\{} & {} \quad +\frac{P((m+1) h,k\Delta \tau )-2P(m h,(k+1)\Delta \tau )+P((m-1)h,k\Delta \tau )}{h^2})ds\Bigg|^2\\{} & {} \quad +4 E \Bigg|\int ^{(k+1)\Delta \tau }_{k\Delta \tau }\left( -P(m h,u)(a-bP(m h,u))+P(m h,k\Delta \tau )(a -bP(m h,(k+1)\Delta \tau ))\right) du\Bigg|^2\\{} & {} \quad + 4c^2E\Bigg|\int ^{(k+1)\Delta \tau }_{k\Delta \tau }\left( \frac{Q(m h,u)P(m h,u)}{P(m h,u)+mQ(m h,u)}- \frac{Q(m h, k \Delta \tau )P(m h,(k+1)\Delta \tau )}{P(m h,k\Delta \tau )+m Q(m h,k\Delta \tau )}\right) du\Bigg|^2 \\{} & {} \quad +4 \eta _1^2E\Bigg|\int ^{(k+1)\Delta \tau }_{k\Delta \tau }\left( -P(m h,u)+P(m h,k\Delta \tau ) \right) d B_1(u)\Bigg|^2. \end{aligned}$$

By using the symmetry property of the Itô’s integral

$$\begin{aligned}{} & {} E|L(P)|_{m,k}-L|_{m,k} P|^2 \le 4\sigma _1^2 E \Bigg|\int ^{(k+1)\Delta \tau }_{k\Delta \tau }(-P_{xx}(m h,u)\\{} & {} \quad +\frac{P((m+1) h,k\Delta \tau )-2P(m h,(k+1)\Delta \tau )+P((m-1)h,k\Delta \tau )}{h^2})ds\Bigg|^2\\{} & {} \quad +4 E \Bigg|\int ^{(k+1)\Delta \tau }_{k\Delta \tau }\left( -P(m h,u)(a-bP(m h,u))+P(m h,k\Delta \tau )(a -bP(m h,(k+1)\Delta \tau ))\right) du\Bigg|^2\\{} & {} \quad + 4c^2E\Bigg|\int ^{(k+1)\Delta \tau }_{k\Delta \tau }\left( \frac{Q(m h,u)P(m h,u)}{P(m h,u)+mQ(m h,u)}- \frac{Q(m h, k \Delta \tau )P(m h,(k+1)\Delta \tau )}{P(m h,k\Delta \tau )+m Q(m h,k\Delta \tau )}\right) du\Bigg|^2 \\{} & {} \quad +4 \eta _1^2 \int ^{(k+1)\Delta \tau }_{k\Delta \tau }E\Bigg|(-P(m h,u)+P(m h,k\Delta \tau ))\Bigg|^2 du. \end{aligned}$$

$E|L(P)|_{m,k}-L|_{m,k}(P)|^2\rightarrow 0$ as $m\rightarrow \infty , q \rightarrow \infty ,$ so the proposed scheme for *P* is consistent with stochastic PDE (2).

Now, to check the consistency of 14 with SPDE (3).

25 $$\begin{aligned} L(Q)|_{m,k}= & {} Q(m h,(k+1)\Delta \tau )-Q(m h,k\Delta \tau )- \sigma _2 \int ^{(k+1)\Delta \tau }_{k\Delta \tau }Q{xx}(m h,u) du \nonumber \\{} & {} + \int ^{(k+1)\Delta \tau }_{k\Delta \tau }d Q(m h,u)du- f \int ^{(k+1)\Delta \tau }_{k\Delta \tau }\frac{Q(m h,u)P(m h,u)}{P(m h,u)+mQ(m h,u)}du \nonumber \\{} & {} - \eta _2 \int ^{(k+1)\Delta \tau }_{k\Delta \tau }Q(m h,u) d B_2(u). \end{aligned}$$

By using the proposed stochastic NSFD scheme on Eq. (3)

26 $$\begin{aligned} L|_{m,k}(Q)= & {} Q(m h,(k+1)\Delta \tau )-Q(m h,k\Delta \tau )\nonumber \\{} & {} -\sigma _2 \Delta \tau \frac{Q((m+1)h,k\Delta \tau )-2Q(m h,(k+1)\Delta \tau )+Q((m-1)h,k\Delta \tau )}{h^2} \nonumber \\{} & {} +d \Delta \tau Q(m h,(k+1)\Delta \tau )- f\Delta \tau \frac{Q(m h, k \Delta \tau )P(m h,k \Delta \tau )}{P(m h,k\Delta \tau )+m Q(m h,k\Delta \tau )}\nonumber \\{} & {} - \eta _2 Q(m h,k\Delta \tau ) (B_2^{(k+1)\Delta \tau }-B_2^{k\Delta \tau }). \end{aligned}$$

Equations (25) and (26) takes the form

$$\begin{aligned}{} & {} E|L(Q)|_{m,k}-L|_{m,k} Q|^2 \le 4\sigma _2^2 E \Bigg|\int ^{(k+1)\Delta \tau }_{k\Delta \tau }(-Q_{xx}(m h,u)\\{} & {} \quad +\frac{Q((m+1) h,k\Delta \tau )-2Q(m h,(k+1) \Delta \tau )+Q((m-1)h,k\Delta \tau )}{h^2})du\Bigg|^2\\{} & {} \quad +4 d^2 E \Bigg|\int ^{(k+1)\Delta \tau }_{k\Delta \tau }\left( Q(m h,u)-Q(m h,(k+1)\Delta \tau )\right) du\Bigg|^2\\{} & {} \quad + 4E\Bigg|\int ^{(k+1)\Delta \tau }_{k\Delta \tau }\left( \frac{-f Q(m h,u)P(m h,u)}{P(m h,u)+mQ(m h,u)}+\frac{f Q(m h, k \Delta \tau )P(m h,k \Delta \tau )}{P(m h,k\Delta \tau )+m Q(m h,k\Delta \tau )}\right) du\Bigg|^2 \\{} & {} \quad +4 \eta _2^2E\Bigg|\int ^{(k+1)\Delta \tau }_{k\Delta \tau }\left( -Q(m h,u)+Q(m h,k\Delta \tau ) \right) d B_2(u)\Bigg|^2. \end{aligned}$$

By using the symmetry property of the Itô’s integral

$$\begin{aligned}{} & {} E|L(Q)|_{m,k}-L|_{m,k} Q|^2 \le 4\sigma _2^2 E \Bigg|\int ^{(k+1)\Delta \tau }_{k\Delta \tau }(-Q_{xx}(m h,u)\\{} & {} \quad +\frac{Q((m+1) h,k\Delta \tau )-2Q(m h,(k+1) \Delta \tau )+Q((m-1)h,k\Delta \tau )}{h^2})du\Bigg|^2\\{} & {} \quad +4 d^2 E \Bigg|\int ^{(k+1)\Delta \tau }_{k\Delta \tau }\left( Q(m h,u)-Q(m h,(k+1)\Delta \tau )\right) du\Bigg|^2\\{} & {} \quad + 4E\Bigg|\int ^{(k+1)\Delta \tau }_{k\Delta \tau }\left( \frac{-f Q(m h,u)P(m h,u)}{P(m h,u)+mQ(m h,u)}+\frac{f Q(m h, k \Delta \tau )P(m h,k \Delta \tau )}{P(m h,k\Delta \tau )+m Q(m h,k\Delta \tau )}\right) du\Bigg|^2\\{} & {} \quad +4 4\eta _2^2 \int ^{(k+1)\Delta \tau }_{k\Delta \tau }E\Bigg|(-Q(m h,u)+Q(m h,k\Delta \tau ))\Bigg|^2 du. \end{aligned}$$

$E|L(Q)|_{m,k}-L|_{m,k} Q|^2\rightarrow 0$ as $m\rightarrow \infty , k \rightarrow \infty ,$ so the proposed scheme for *Q* is consistent with stochastic PDE (3). $\square$

### Stability of the scheme-I

#### Theorem 4

If $|1+ a \Delta \tau - 4D_1\sin ^2(\frac{\Delta x \eta }{2})|^2\le 1$, and $|1-d \Delta \tau - 4D_2\sin ^2(\frac{\Delta x \eta }{2})|^2\le 1$, then the proposed SFE for *P*(*x*, *t*) and *Q*(*x*, *t*) is stable with $(k+1)\Delta \tau =T$.

#### Proof

As the given technique is used on linear equations, Eq. (11) is linearized as follows

$$\begin{aligned} P_m^{k+1}=D_1 (P_{m+1}^k+P_{m-1}^k)+(1-2D_1)P_m^k+a \Delta \tau P_m^k+\eta _1 P_m^k \left( B^{(k+1)\Delta \tau }-B^{k\Delta \tau }\right) , \end{aligned}$$

by using Eq. (16), the above equation takes the following form,

$$\begin{aligned}{} & {} \frac{1}{\sqrt{2\pi }}\int _{\frac{-\pi }{\Delta x}}^{\frac{\pi }{\Delta x}}e^{\iota m\Delta x \eta } \hat{P}^{k+1}_{m}(\eta )d(\eta )=\frac{1}{\sqrt{2\pi }}\int _{\frac{-\pi }{\Delta x}}^{\frac{\pi }{\Delta x}}\bigg (1-2D_1+a \Delta \tau + D_1e^{\iota \Delta x \eta }+D_1e^{-\iota \Delta x \eta }\\{} & {} \quad +\eta _1 \left( B_1^{(k+1)\Delta \tau }-B_1^{k\Delta \tau }\right) \bigg )e^{\iota m\Delta x \eta } \hat{P}^{k}_{m}(\eta )d(\eta ),\\{} & {} \hat{P}^{k+1}_{m}(\eta )=\bigg (1-2D_1+a \Delta \tau + D_1e^{\iota \Delta x \eta }+D_1e^{-\iota \Delta x \eta }+\eta _1 \left( B_1^{(k+1)\Delta \tau }-B_1^{k\Delta \tau }\right) \bigg ) \hat{P}^{k}_{m}(\eta ),\\{} & {} \hat{P}^{k+1}_{m}(\eta )=\bigg (1-2D_1+a \Delta \tau + 2D_1- 4D_1\sin ^2(\frac{\Delta x \eta }{2})+\eta _1 \left( B_1^{(k+1)\Delta \tau }-B_1^{k\Delta \tau }\right) \bigg ) \hat{P}^{k}_{m}(\eta ). \end{aligned}$$

So, the amplification factor takes the form

$$\begin{aligned} g(\eta \Delta x, \Delta x, \Delta t)=\bigg (1+a \Delta \tau - 4D_1\sin ^2(\frac{\Delta x \eta }{2})+\eta _1 \left( B_1^{(k+1)\Delta \tau }-B_1^{k\Delta \tau }\right) \bigg ) \end{aligned}$$

As the Wiener process is independent of the from the state of state variable *P*(*x*, *t*), the amplification factor takes the form

$$\begin{aligned} E\left| g(\eta \Delta x, \Delta x, \Delta t)\right| ^2 \le \bigg |1+a \Delta \tau - 4D_1\sin ^2(\frac{\Delta x \eta }{2})\bigg |^2+|\eta _1|^2 \Delta \tau \end{aligned}$$

if $$|1+a \Delta \tau - 4D_1\sin ^2(\frac{\Delta x \eta }{2})|^2\le 1$$, then

$$\begin{aligned} E\left| g(\eta \Delta x, \Delta x, \Delta t)\right| ^2 \le 1+|\eta _1|^2 \Delta \tau , \end{aligned}$$

where, $|\eta _1|^2=\chi$. So, given for (2) is stable. Now, for the stability of Eqs. (3), (12) is linearized as follow

27 $$\begin{aligned} Q_m^{k+1}= D_2 (Q_{m+1}^k+Q_{m-1}^k)+(1-2D_2- d \Delta \tau )Q_m^k+ \eta _2 Q_m^k \left( B_2^{(k+1)\Delta \tau }-B_2^{k\Delta \tau }\right) , \end{aligned}$$

As the given technique is used on linear equations, Eq. (12) is linearized as follows by using Eq. (16), the above equation takes the following form,

$$\begin{aligned}{} & {} \frac{1}{\sqrt{2\pi }}\int _{\frac{-\pi }{\Delta x}}^{\frac{\pi }{\Delta x}}e^{\iota m\Delta x \eta } \hat{Q}^{k+1}_{m}(\eta )d(\eta )=\frac{1}{\sqrt{2\pi }}\int _{\frac{-\pi }{\Delta x}}^{\frac{\pi }{\Delta x}}\bigg (1-2D_2-d \Delta \tau + D_2e^{\iota \Delta x \eta }+D_2e^{-\iota \Delta x \eta }\\{} & {} \quad +\eta _2 \left( B_2^{(k+1)\Delta \tau }-B_2^{k\Delta \tau }\right) \bigg )e^{\iota m\Delta x \eta } \hat{Q}^{k}_{m}(\eta )d(\eta ),\\{} & {} \hat{Q}^{k+1}_{m}(\eta )=\bigg (1-2D_2-d \Delta \tau + D_2e^{\iota \Delta x \eta }+D_2e^{-\iota \Delta x \eta }+\eta _2 \left( B_2^{(k+1)\Delta \tau }-B_2^{k\Delta \tau }\right) \bigg ) \hat{Q}^{k}_{m}(\eta ),\\{} & {} \hat{P}^{k+1}_{m}(\eta )=\bigg (1-2D_2-d \Delta \tau + 2D_2- 4D_2\sin ^2(\frac{\Delta x \eta }{2})+\eta _2 \left( B_2^{(k+1)\Delta \tau }-B_2^{k\Delta \tau }\right) \bigg ) \hat{P}^{k}_{m}(\eta ). \end{aligned}$$

So, the amplification factor takes the form

$$\begin{aligned} g(\eta \Delta x, \Delta x, \Delta t)=\bigg (1-d \Delta \tau - 4D_2\sin ^2(\frac{\Delta x \eta }{2})+\eta _2 \left( B_2^{(k+1)\Delta \tau }-B_2^{k\Delta \tau }\right) \bigg ). \end{aligned}$$

As the Wiener process is independent of the from the state of state variable *P*(*x*, *t*), the amplification factor takes the form

$$\begin{aligned} E\left| g(\eta \Delta x, \Delta x, \Delta t)\right| ^2 \le \bigg |1-d \Delta \tau - 4D_2\sin ^2(\frac{\Delta x \eta }{2})\bigg |^2+|\eta _2|^2 \Delta \tau , \end{aligned}$$

if $$|1-d \Delta \tau - 4D_2\sin ^2(\frac{\Delta x \eta }{2})|^2\le 1$$, then

$$\begin{aligned} E\left| g(\eta \Delta x, \Delta x, \Delta t)\right| ^2 \le 1+|\eta _2|^2 \Delta \tau , \end{aligned}$$

where, $|\eta _2|^2=\chi$. So, the given scheme for (3) is stable. $\square$

### Stability of the scheme-II

#### Theorem 5

The proposed stochastic NSFD scheme for *P*(*x*, *t*) and *Q*(*x*, *t*) is unconditionally stable in the mean square sense.

#### Proof

As the given technique is used on linear equations, Eq. (13) is linearized as follows

$$\begin{aligned} (1+2D_1)P_m^{k+1}=D_1 (P_{m+1}^k+P_{m-1}^k)+P_m^k+a \Delta \tau P_m^k+\eta _1 P_m^k \left( B_1^{(k+1)\Delta \tau }-B_1^{k\Delta \tau }\right) . \end{aligned}$$

By using Eq. (16), the above equation takes the following form

$$\begin{aligned}{} & {} \frac{1}{\sqrt{2\pi }}\int _{\frac{-\pi }{\Delta x}}^{\frac{\pi }{\Delta x}}e^{\iota m\Delta x \eta }(1+2D_1) \hat{P}^{k+1}_{m}(\eta )d(\eta )=\frac{1}{\sqrt{2\pi }}\int _{\frac{-\pi }{\Delta x}}^{\frac{\pi }{\Delta x}}\bigg (1+a \Delta \tau + D_1e^{\iota \Delta x \eta }+D_1e^{-\iota \Delta x \eta }\\{} & {} \quad +\eta _1 \left( B_1^{(k+1)\Delta \tau }-B_1^{k\Delta \tau }\right) \bigg )e^{\iota m\Delta x \eta } \hat{P}^{k}_{m}(\eta )d(\eta ),\\{} & {} \quad (1+2D_1)\hat{P}^{k+1}_{m}(\eta )=\bigg (1+a \Delta \tau + D_1e^{\iota \Delta x \eta }+D_1e^{-\iota \Delta x \eta }+\eta _1 \left( B_1^{(k+1)\Delta \tau }-B_1^{k\Delta \tau }\right) \bigg ) \hat{P}^{k}_{m}(\eta ),\\{} & {} \quad (1+2D_1)\hat{P}^{k+1}_{m}(\eta )=\bigg (1+a \Delta \tau + 2D_1- 4D_1\sin ^2(\frac{\Delta x \eta }{2})+\eta _1 \left( B_1^{(k+1)\Delta \tau }-B_1^{k\Delta \tau }\right) \bigg ) \hat{P}^{k}_{m}(\eta ). \end{aligned}$$

So, the amplification factor takes the form

$$\begin{aligned} g(\eta \Delta x, \Delta x, \Delta t)=\frac{1+2D_1+a \Delta \tau - 4D_1\sin ^2(\frac{\Delta x \eta }{2}}{1+2D_1}+\frac{\eta _1 \left( B_1^{(k+1)\Delta \tau }-B_1^{k\Delta \tau }\right) }{1+2D_1}. \end{aligned}$$

As the Wiener process is independent of the from the state of state variable *P*(*x*, *t*), the amplification factor takes the form

$$\begin{aligned} E\left| g(\eta \Delta x, \Delta x, \Delta t)\right| ^2 \le \bigg |\frac{1+2D_1+a \Delta \tau - 4D_1\sin ^2(\frac{\Delta x \eta }{2}}{1+2D_1}\bigg |^2+\bigg |\frac{\eta _1}{1+2D_1}\bigg |^2 \Delta \tau . \end{aligned}$$

$$\bigg |\frac{1+2D_1+a \Delta \tau - 4D_1\sin ^2(\frac{\Delta x \eta }{2}}{1+2D_1}\bigg |^2\le 1$$, and

$$\begin{aligned} E\left| g(\eta \Delta x, \Delta x, \Delta t)\right| ^2 \le 1+\bigg |\eta _1\bigg |^2 \Delta \tau , \end{aligned}$$

where, $\bigg |\frac{\eta _1}{1+2D_1}\bigg |^2=\chi$. So, given for (2) is stable. Now, for the stability of Eq. (3), Eq. (14) is linearized as follow

28 $$\begin{aligned} (1+2D_2+d \Delta \tau )Q_m^{k+1}= D_2 (Q_{m+1}^k+Q_{m-1}^k)+Q_m^k+ \eta _2 Q_m^k \left( B_2^{(k+1)\Delta \tau }-B_2^{k\Delta \tau }\right) . \end{aligned}$$

By using Eq. (16), the above equation takes the following form

$$\begin{aligned}{} & {} \frac{1}{\sqrt{2\pi }}\int _{\frac{-\pi }{\Delta x}}^{\frac{\pi }{\Delta x}}e^{\iota m\Delta x \eta } (1+2D_2+d \Delta \tau ) \hat{Q}^{k+1}_{m}(\eta )d(\eta )=\frac{1}{\sqrt{2\pi }}\int _{\frac{-\pi }{\Delta x}}^{\frac{\pi }{\Delta x}}\bigg (1+ D_2e^{\iota \Delta x \eta }+D_2e^{-\iota \Delta x \eta }\\{} & {} \quad +\eta _2 \left( B_2^{(k+1)\Delta \tau }-B_2^{k\Delta \tau }\right) \bigg )e^{\iota m\Delta x \eta } \hat{Q}^{k}_{m}(\eta )d(\eta ).\\{} & {} \quad (1+2D_2+d \Delta \tau )\hat{Q}^{k+1}_{m}(\eta )=\bigg (1+D_2e^{\iota \Delta x \eta }+D_2e^{-\iota \Delta x \eta }+\eta _2 \left( B_2^{(k+1)\Delta \tau }-B_2^{k\Delta \tau }\right) \bigg ) \hat{Q}^{k}_{m}(\eta ).\\{} & {} \quad (1+2D_2+d \Delta \tau )\hat{P}^{k+1}_{m}(\eta )=\bigg (1+ 2D_2- 4D_2\sin ^2(\frac{\Delta x \eta }{2})+\eta _2 \left( B_2^{(k+1)\Delta \tau }-B_2^{k\Delta \tau }\right) \bigg ) \hat{P}^{k}_{m}(\eta ). \end{aligned}$$

So, the amplification factor takes the form

$$\begin{aligned} g(\eta \Delta x, \Delta x, \Delta t)=\frac{1+2D_2- 4D_2\sin ^2(\frac{\Delta x \eta }{2})}{(1+2D_2+d \Delta \tau )}+\frac{\eta _2 (B_2^{(k+1)\tau }-B_2^{k\tau })}{1+2D_2+d \Delta \tau }. \end{aligned}$$

As the Wiener process is independent of the from the state of state variable *P*(*x*, *t*), the amplification factor takes the form

$$\begin{aligned} E\left| g(\eta \Delta x, \Delta x, \Delta t)\right| ^2 \le |\frac{1+2D_2- 4D_2\sin ^2(\frac{\Delta x \eta }{2})}{1+2D_2+d \Delta \tau }|^2+|\frac{\eta _2}{1+2D_2+d \Delta \tau }|^2 \Delta \tau . \end{aligned}$$

as $$|\frac{1+2D_2- 4D_2\sin ^2(\frac{\Delta x \eta }{2})}{(1+2D_2+d \Delta \tau )}|^2\le 1$$, then

$$\begin{aligned} E\left| g(\eta \Delta x, \Delta x, \Delta t)\right| ^2 \le 1+|\frac{\eta _2}{1+2D_2+d \Delta \tau }|^2 \Delta \tau , \end{aligned}$$

where, $|\frac{\eta _2}{1+2D_2+d \Delta \tau }|^2=\chi$. So, the given scheme is unconditionally stable for (14). $\square$

## Results and discussion

### Problem 1

$$\begin{aligned} \frac{\partial P}{\partial t}= & {} \sigma _1\frac{\partial ^2 P}{\partial x^2}+ P(a-bP)-\frac{cPQ}{mQ+P}+\eta _1 P \dot{B}(t),\\ \frac{\partial Q}{\partial t}= & {} \sigma _2 \frac{\partial ^2 Q}{\partial x^2}-dQ +\frac{fPQ}{mQ+P}+\eta _2 Q \dot{B}(t). \end{aligned}$$

with initial conditions^10^,

$$\begin{aligned} P(x,0)= 0.6, \qquad Q(x,0)= 0.4, \end{aligned}$$

and having homogeneous Neumann boundary conditions. The system of Eqs. (2) and (3) have two equilibriums, one is predator free point (1, 0) and second is coexistence point $(P^*,Q^*)$, where $P^*=\frac{(am-c)f+cd}{bmf}$ and $Q^*=\frac{(f-d)(amf-cf+cd)}{bdm^2f}$. The coexistence equilibrium point is stable or unstable if $(c-mf)d^2-(c-ma-md)f^2 >0$ or $<0$^10^.

**Figure 1:**
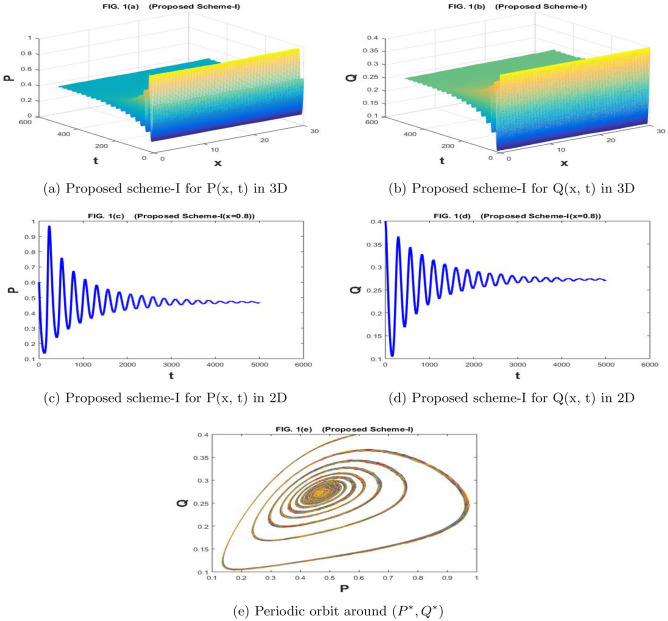
The subfig (**a** and **c**) and (**b** and **d**) represents the numerical approximation of *P*(*x*, *t*), and *Q*(*x*, *t*) respectively by proposed scheme-I. The subfig (**e**) represents the periodic orbit around $(P^*,Q^*)$.

**Figure 2:**
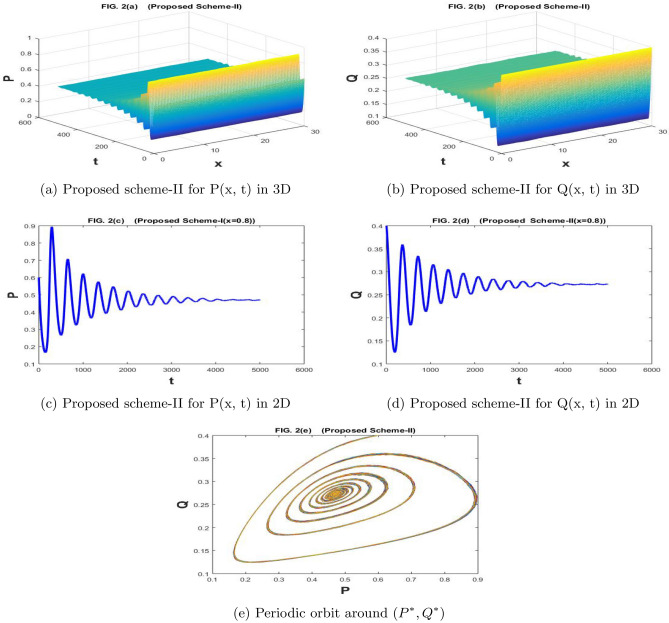
The subfig (**a** and **c**) and (**b** and **d**) represents the numerical approximation of *P*(*x*, *t*), and *Q*(*x*, *t*) respectively by proposed scheme-II. The subfig (**e**) represents the periodic orbit around $(P^*,Q^*)$.

The Fig. 1a–e are drawn by the proposed scheme-I. The Fig. 2a–e are drawn by the proposed scheme-II. We choose the values of the parameters as follows for Figs. 1a–e) and 2a–e) $a=1.1$, $\sigma _1=\sigma _2=0.1$, $m=1$, $c=2.1$, $b=0.7$, $f=0.79$, $d=0.5$,$n=100$,$h=30/n$, $N=5000$, $\eta _1=\eta _2=0.01$, and $k=500/N$, then coexistence equilibrium point $(P^*,Q^*)$ has value (0.4699, 0.2719) by proposed scheme-I and scheme-II . The solution is stable and converging to $(P^*, Q^*)$ and it is depicted graphically in Figs. 1e and 2e.

**Figure 3:**
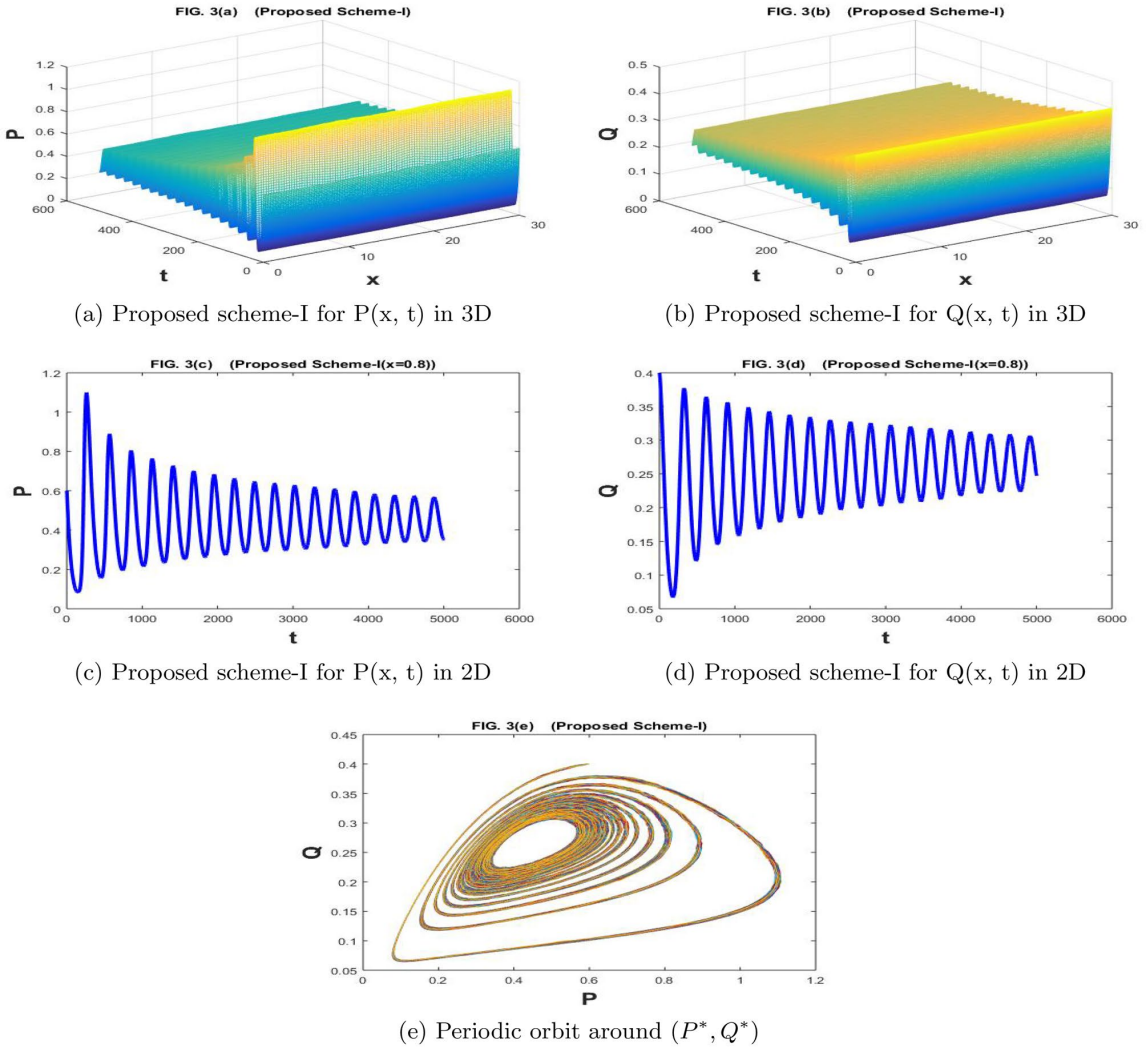
The subfig (**a** and **c**) and (**b** and **d**) represents the numerical approximation of *P*(*x*, *t*), and *Q*(*x*, *t*) respectively by proposed scheme-I. The subfig (**e**) represents the periodic orbit around $(P^*,Q^*)$.

**Figure 4:**
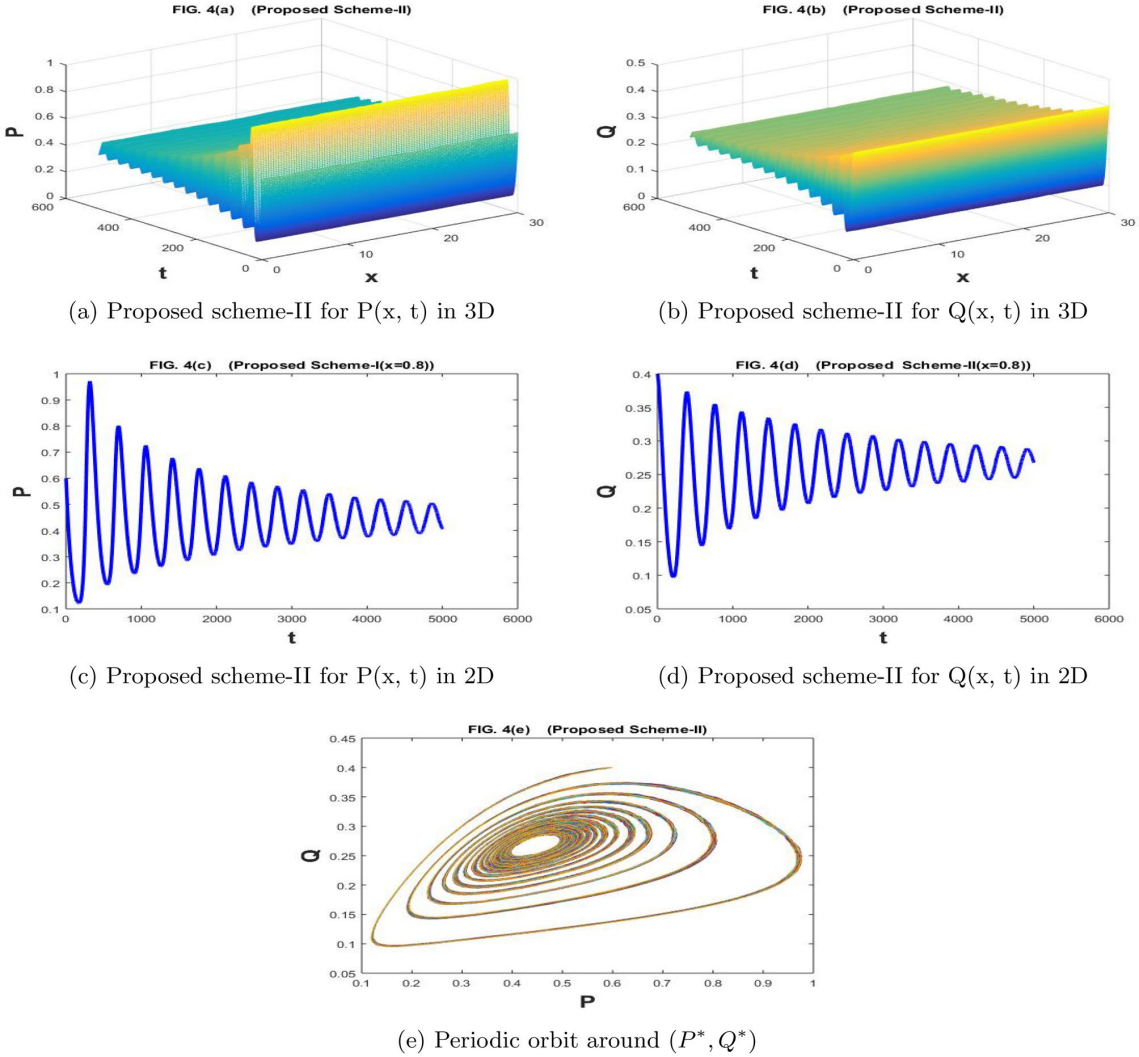
The subfig (**a** and **c**) and (**b** and **d**) represents the numerical approximation of *P*(*x*, *t*), and *Q*(*x*, *t*) respectively by proposed scheme-II. The subfig (**e**) represents the periodic orbit around $(P^*,Q^*)$.

The Fig. 3a–e are drawn by the proposed scheme-I. The Fig. 4a–e are drawn by the proposed scheme-II. We have taken the values of the parameters as $a=1.1$, $\sigma _1=\sigma _2=1$, $m=1$, $c=2.1$, $b=0.7$, $f=0.80$, $d=0.5$,$n=100$,$h=30/n$, $N=5000$, $\eta _1=\eta _2=0.01$, and $k=500/N$. If we increase the value of *f* gradually and it passes through the bifurcation values, the behavior of our system changes and it becomes unstable in Figs. 3a–e and 4a–e the population densities *P* and *Q* have a periodic orbit around $(P^*, Q^*)$. Both prey and predator have oscillations behavior, which can be observed in 2*D* graphs of Figs. 3b, d, and 4b, d.

**Figure 5:**
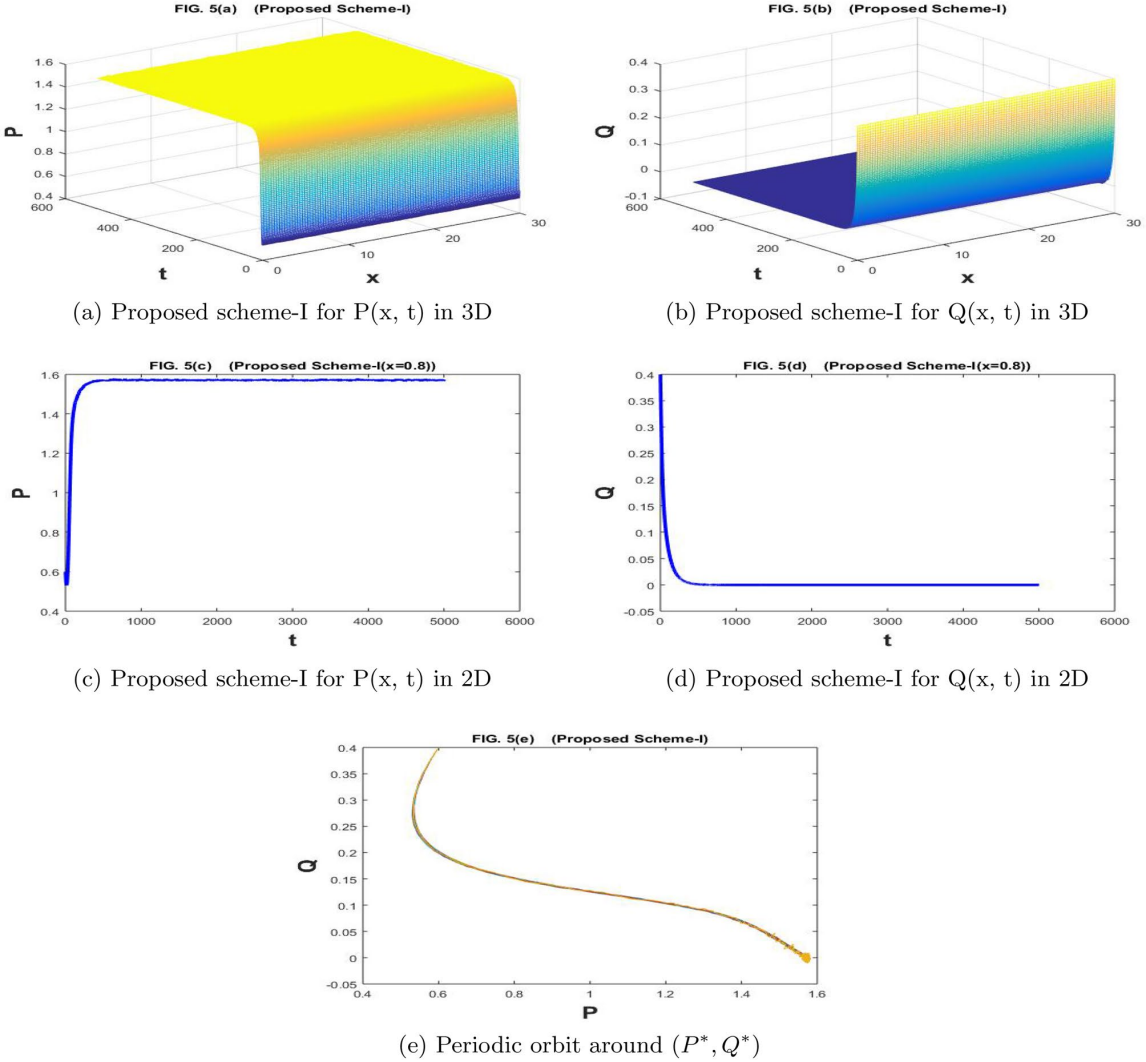
The subfig (**a** and **c**) and (**b** and **d**) represents the numerical approximation of *P*(*x*, *t*), and *Q*(*x*, *t*) respectively by proposed scheme-I. The subfig (**e**) represents the periodic orbit around $(P^*,Q^*)$.

**Figure 6:**
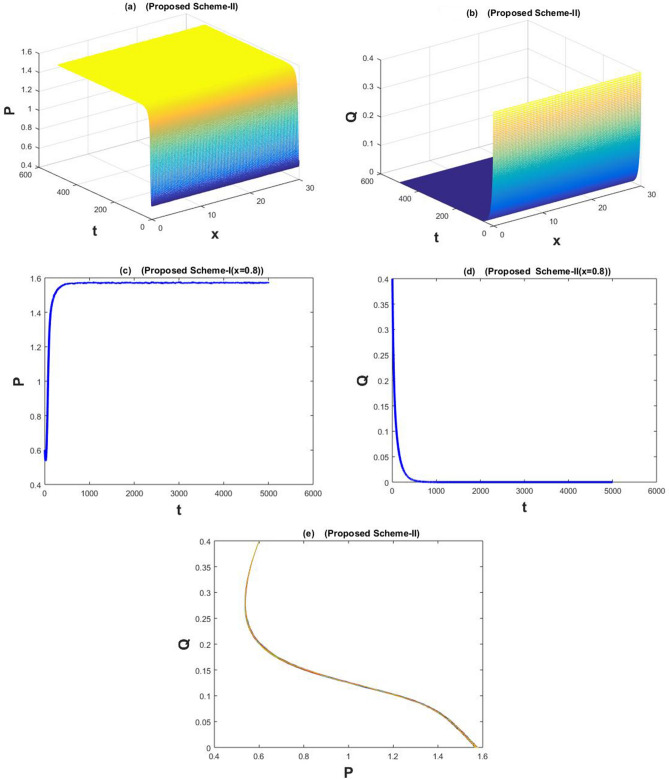
The subfig (**a** and **c**) and (**b** and **d**) represents the numerical approximation of *P*(*x*, *t*), and *Q*(*x*, *t*) respectively by proposed scheme-II. The subfig (**e**) represents the periodic orbit around $(P^*,Q^*)$.

The Fig. 5a–e are drawn by the proposed scheme-I. The Fig. 6a–e are drawn by the proposed scheme-II. We have taken the values of the parameters as $a=1.1$, $\sigma _1=\sigma _2=0.1$, $m=1$, $c=2.1$, $b=0.7$, $f=0.4$, $d=0.5$,$n=100$,$h=30/n$, $N=5000$, $\eta _1=\eta _2=0.01$, and $k=50/N$, in Fig. 5a–e and 6a–e. The population densities *P* and *Q* approach a predator-free point i.e., (1,0).

**Figure 7:**
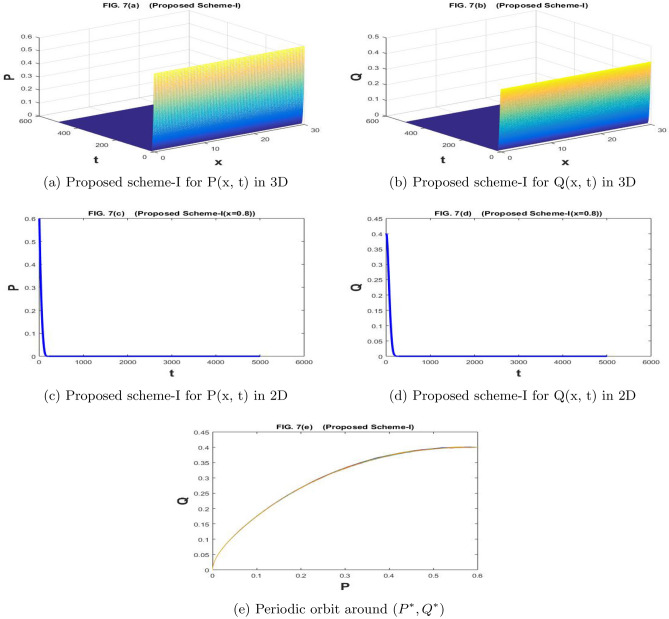
The subfig (**a** and **c**) and (**b** and **d**) represents the numerical approximation of *P*(*x*, *t*), and *Q*(*x*, *t*) respectively by proposed scheme-I. The subfig (**e**) represents the periodic orbit around $(P^*,Q^*)$.

**Figure 8:**
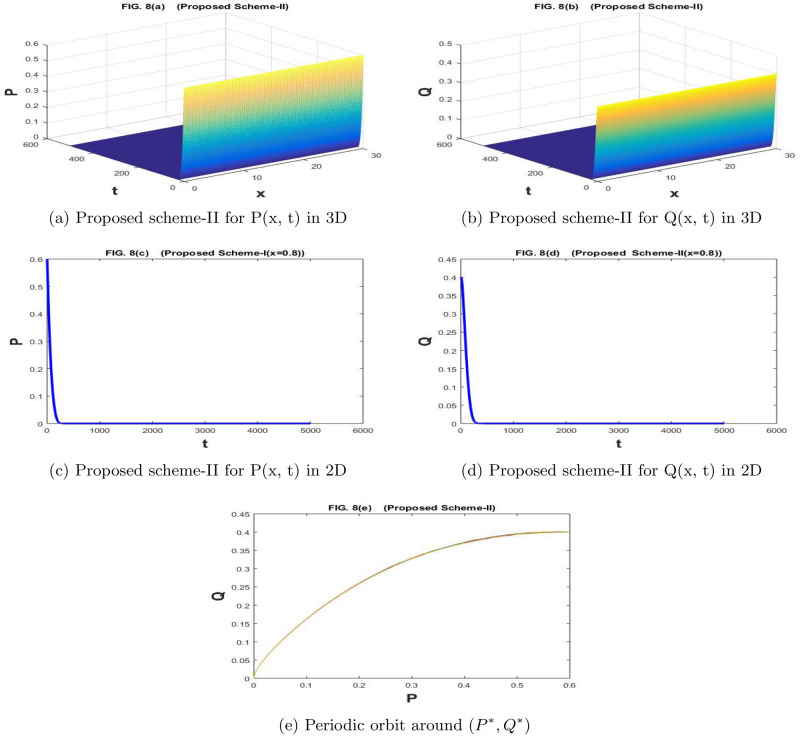
The subfig (**a** and **c**) and (**b** and **d**) represents the numerical approximation of *P*(*x*, *t*), and *Q*(*x*, *t*) respectively by proposed scheme-II. The subfig (**e**) represents the periodic orbit around $(P^*,Q^*)$.

The Fig. 7a–e are drawn by the proposed scheme-I. The Fig. 8a–e are drawn by the proposed scheme-II. By increasing the values of parameter *f*, we have reached a point where both prey and predator populations are extinct. So, parameter *f* can be used as the control parameter of the prey-predator model. It can be used to change the behavior of the model from stable to unstable behavior and from unstable behavior to the extinction of both species. We choose the values of the parameters as $a=1.1$, $\sigma _1=\sigma _2=0.1$, $m=1$, $c=2.1$, $b=0.7$, $f=0.84$, $d=0.5$,$n=100$,$h=30/n$, $N=5000$, $\eta _1=\eta _2=0.01$, and $k=500/N$. The population densities *P* and *Q* approach (0,0).

**Figure 9:**
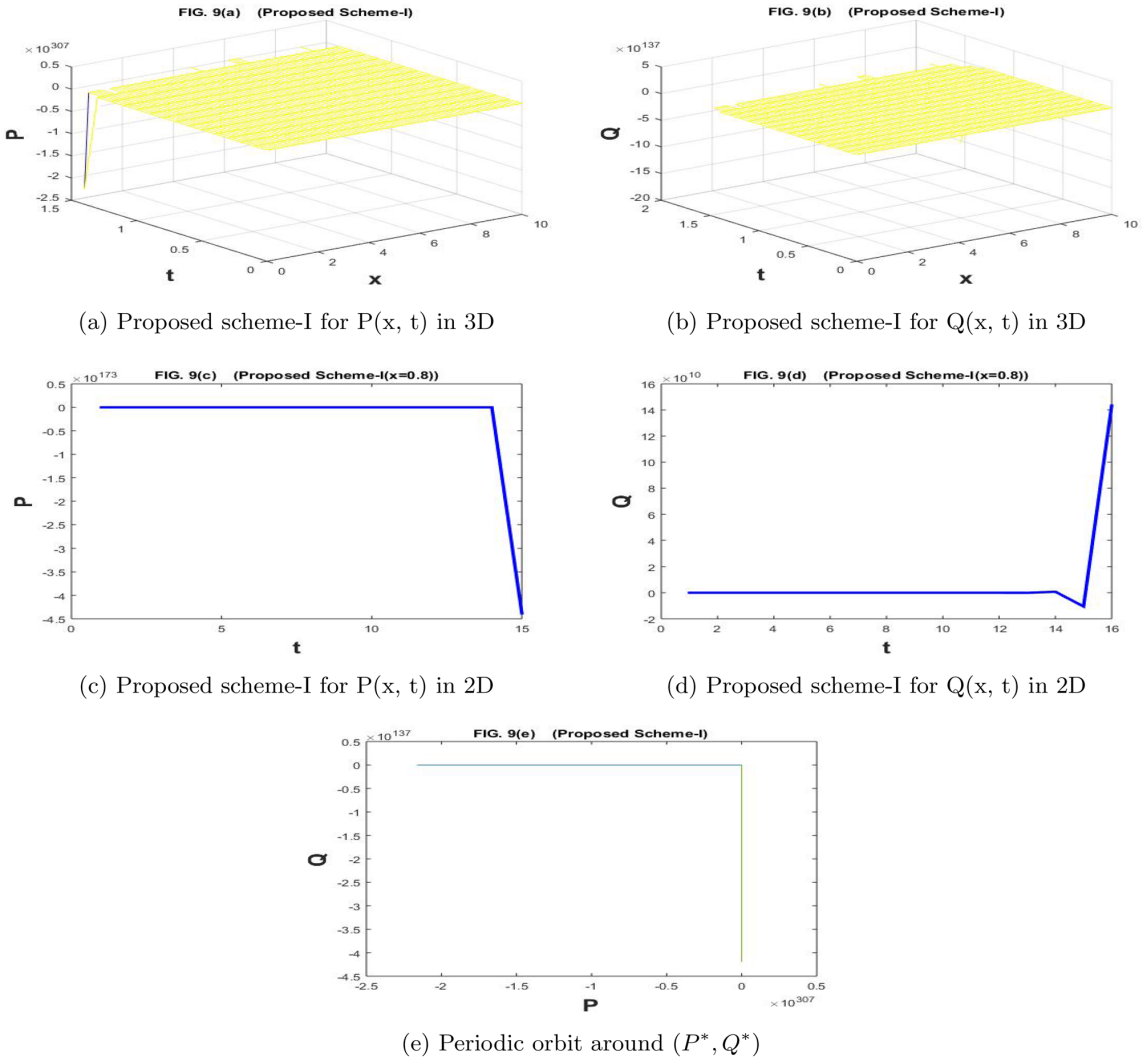
The subfig (**a** and **c**) and (**b** and **d**) represents the numerical approximation of *P*(*x*, *t*), and *Q*(*x*, *t*) respectively by proposed scheme-I. The subfig (**e**) represents the periodic orbit around $(P^*,Q^*)$.

**Figure 10:**
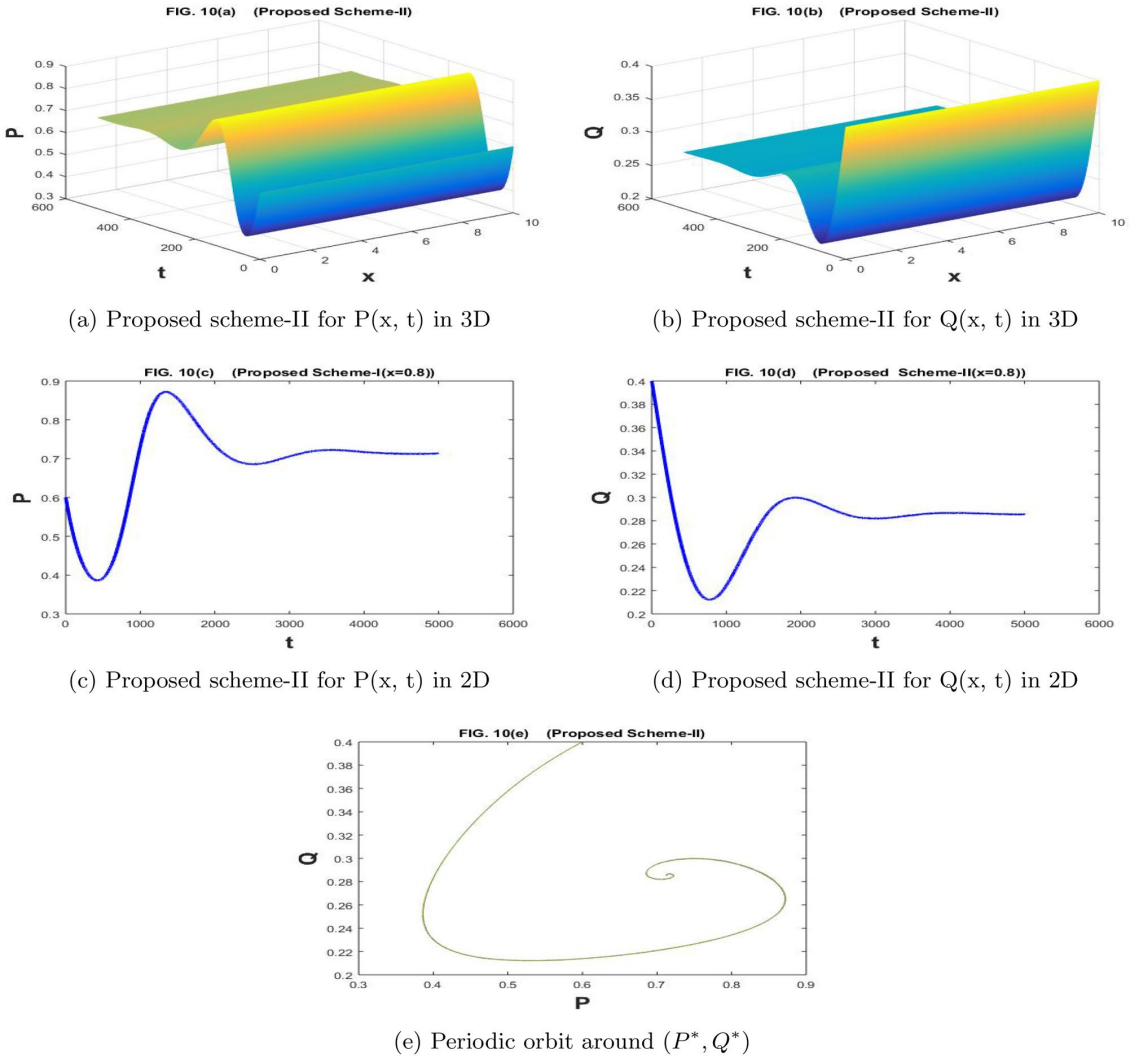
The subfig (**a** and **c**) and (**b** and **d**) represents the numerical approximation of *P*(*x*, *t*), and *Q*(*x*, *t*) respectively by proposed scheme-II. The subfig (**e**) represents the periodic orbit around $(P^*,Q^*)$.

The Fig. 9a–e are drawn by the proposed scheme-I. The Fig. 10a–e are drawn by the proposed scheme-II. We have chosen the values of the parameters as $a=1.1$, $\sigma _1=\sigma _2=1$, $m=1$, $c=2.1$, $b=0.7$, $f=0.7$, $d=0.5$,$n=60$,$h=10/n$, $N=5000$, $\eta _1=\eta _2=0.01$, and $k=500/N$, in Fig. 9a–e and 10a–e. For given values of the parameters, scheme-I showed divergence and negative behavior but scheme-II has convergent and positive behavior. The proposed scheme II preserves the true traits of the underlying model. Such models cannot possess negative values. So, scheme II can be recommended for the solution of such models.

In Fig. 9a–e the numerical solution is provided by the stochastic forward Euler scheme and it has divergent as well as negative behavior for the given values of the parameters. Such solutions are not permitted for the papulation dynamical model because the population may attain a minimum value of zero and it can never attain negative values. A solution that preserves the positive behavior for the whole domain is preferable because the positivity of the solutions is a basic property for the population dynamical models. The proposed stochastic non-standard finite difference scheme is applied for the underlying model and we gave obtained the convergent as well positive solutions for the whole domain.

## Conclusion

The ratio-dependent prey-predator model under the influence of time noise has been numerically investigated by two novel schemes. The existence of the solutions is guaranteed by using the fixed point theory with a priori estimates. Both schemes are consistent with the systems of the equations in the mean square sense. The stability is shown by Von-Neumann criteria. The proposed scheme-I is conditionally stable and conditions are derived. The proposed scheme II is stable for the whole domain. We have gained a coexistence equilibrium point for the different values of the parameters. By increasing the values of the conversion rate *f*, the systems change the behavior from stable to unstable. When we further increased the values of the parameter *f*, the population densities become extinct. So, conversion rate *f* played a key role in the system obtaining desired results. For specified values of the parameters, we have also gained a predator-free point. The graphical behavior of a test problem for different values of the parameters is drawn which depicts the efficacy of the schemes. Our numerical solutions are well accurate to the solutions available in the literature. As population densities have positivity, so there must be a scheme that possesses such properties. So, the proposed stochastic non-standard finite difference scheme is preferred which preserves all properties. Hopefully, these results will motivate the researchers to consider the stochastic prey-predator model and analyze them.

## Data Availability

All data generated or analysed during this study are included in this published article.
